# Ultrasonic-assisted extraction of luteolin from peanut shells using ionic liquid and its molecular mechanism

**DOI:** 10.1016/j.ultsonch.2025.107228

**Published:** 2025-01-12

**Authors:** Liwei Niu, Siwen Zhang, Xiaoyu Si, Yuhan Fang, Shuang Wang, Lulu Li, Zunlai Sheng

**Affiliations:** a College of Veterinary Medicine, Northeast Agricultural University, Harbin 150030, PR China; b Heilongjiang Key Laboratory for Animal Disease Control and Pharmaceutical Development, Harbin 150030, PR China

**Keywords:** Peanut shells, Luteolin, Ionic liquids, Ultrasound-assisted extraction, Interaction energy, Extraction kinetics

## Abstract

This study investigates the potential of ionic liquids (ILs) as sustainable solvents in ultrasonic-assisted extraction (UAE) to efficiently recover luteolin from peanut shells. Among the range of ILs tested, 1-butyl-3-methylimidazolium tetrafluoroborate stood out as the most effective solvent, achieving the highest extraction yield. Single-factor experiments were conducted to analyze the effects of ultrasonic power, extraction time, extraction temperature, IL concentration, and solid-to-liquid ratio on extraction efficiency. Further optimization of the extraction conditions was performed using response surface methodology and neural network analysis, resulting in a significantly enhanced luteolin yield of 3.71 ± 0.06 mg/g. Interaction energy analyses were conducted to elucidate the interactions between ILs and luteolin, confirming the experimental findings and highlighting the strongest interaction energy between 1-butyl-3-methylimidazolium tetrafluoroborate and luteolin. A kinetic model for luteolin extraction was developed, demonstrating that the extraction process follows a second-order rate model, where the extraction rate is directly proportional to the square of the concentration difference between luteolin and the solvent. The outcomes of this research present an efficient protocol for luteolin extraction and provide novel insights into the application of UAE in extracting natural products.

## 1 Introduction

China is a leading global producer of peanuts, accounting for approximately 30 % of the total weight of peanut pods during the shelling process. Despite this, only a small portion of peanut shells is processed into animal feed or utilized as chemical raw materials. The majority is either used as fuel or discarded as waste, resulting in significant environmental pollution and a considerable loss of biomass resources [1]. Peanut shells are not only rich in crude fiber, protein, fats, carbohydrates, and minerals but also contain various flavonoids, including luteolin, eriodictyol, and diosmetin[2], [3].

Luteolin is recognized for its wide array of biological attributes, which encompass antioxidant, antibacterial, anti-inflammatory, cardiovascular protection, and neuroprotective effects [4], [5], [6], [7]. Consequently, luteolin holds significant promise for applications in various sectors, such as food, pharmaceuticals, health products, and cosmetics. Additionally, pharmacological studies and clinical trials indicate its efficacy in inhibiting cancer cell proliferation and reducing blood lipid levels [8], [9], [10]. Therefore, this study aims to utilize peanut shells as a raw material for the green extraction of luteolin, which is crucial for subsequent clinical investigations into its biological activities.

The current methods for extracting luteolin predominantly include ethanol–water solvent reflux extraction, alkaline dilute alcohol extraction, supercritical fluid extraction, enzymatic extraction, and microporous resin adsorption [11], [12], [13], [14]. However, these traditional methods often present challenges, such as high equipment costs, complex operational requirements, and the potential degradation of active components. Thus, developing a luteolin extraction process that is both effective and environmentally benign continues to be a major issue.

Ultrasound-assisted extraction (UAE) is an innovative technique for extracting bioactive compounds. The application of ultrasound effectively disrupts cell walls within a short duration, facilitating the release of target substances. This method significantly reduces operational time, streamlines procedures, minimizes solvent consumption and temperature requirements, and lowers energy input [15]. Consequently, UAE is frequently recognized as an “environmentally friendly” or “green” extraction technology that minimizes adverse effects on active components, decreases environmental pollution, and ensures safety [16].

In the pursuit of environmentally sustainable and efficient extraction methods, the choice of solvent is as critical as the extraction technology itself. Traditional organic solvents often exhibit high volatility, considerable toxicity, and adverse environmental impacts. Consequently, it is essential to identify a solvent that not only enhances extraction efficiency but also minimizes ecological damage.

In this regard, ionic liquids (ILs) have become green solvents that show promise and need investigation. ILs are composed exclusively of organic cations paired with either inorganic or organic anions, and they remain in a liquid state at ambient temperatures. They are considered environmentally benign solvents due to their low vapor pressure, low volatility, and good thermal stability [17]. Imidazolium-based ILs are particularly favored due to their capacity to enhance extraction yield through the high acidity of their cation structures. Moreover, ILs can be structurally adjusted to address the hydrophilicity or hydrophobicity of target active ingredients in plant materials, thereby increasing the solubility of these compounds in extraction solutions. Research has indicated that combining with ultrasonic techniques further increases extraction efficiency [18], [19], [20]. When combined with ultrasonic methods, extraction efficiency is further enhanced. Therefore, ionic liquid ultrasound-assisted extraction (ILs-UAE) emerges as a highly effective extraction technique; the synergistic effects of ultrasonic cavitation and thermal energy facilitate the dissolution of active components, reduce extraction time and energy consumption, and ultimately boost the yield of valuable substances [21]. In comparison, other green solvents such as supercritical fluids and aqueous solvents, while having their advantages, may not perform as excellently as ionic liquids in certain aspects. For example, supercritical fluids [22] have high solubility, but their equipment investment and maintenance costs are high; whereas water solvents [23] may not achieve ideal extraction results in certain cases. Therefore, overall, ionic liquids, as a new type of green solvent, have shown significant advantages and potential in the extraction of luteolin.

The luteolin from peanut shells is extracted in this study using ILs-UAE. To identify the ideal extraction process conditions, response surface methodology (RSM) and artificial neural networks optimized using genetic algorithms (ANN-GA) were used in conjunction with the single-factor analysis. Additionally, interaction energy analysis was utilized to explore the interaction mechanisms between ILs and luteolin. Discussing the kinetic processes under optimal extraction conditions provides theoretical and practical support for developing and utilizing luteolin from peanut shells.

## 2 Materials and methods

### 2.1 Materials and Instruments

Peanuts (Cultivar: Luhua No.4) were purchased from a local supermarket. The shells were manually extracted and subsequently dried in an oven at 80°C for 24 h. Following drying, the peanut shells were ground and sieved using a 40-mesh pharmaceutical sieve to ensure uniformity in particle size, and subsequently stored in a plastic bag for future experimental use. Luteolin with ≥98 % purity was procured from Shanghai Maclin Biochemical Technology Co., Ltd. (Lot: L107329). HPLC-grade acetonitrile and formic acid were obtained from Kemio Chemical Reagents Co., Ltd. (Tianjin). Deionized water was produced using a Milli-Q Academic water purification system, and all other analytical-grade reagents were sourced from Aladdin Reagent Co., Ltd. (Shanghai, China).

A KQ-250DB ultrasonic generator (Kunshan, Jiangsu, China), with a maximum output power of 250 W, was employed for the sonochemical reactions in this study. The device consists of a rectangular reaction tank measuring 23.5 cm × 13.3 cm × 10.3 cm. Annealed ultrasonic transducers operating at a frequency of 50 kHz are attached to the vessel's base. For HPLC analysis of luteolin, a Shimadzu LC-20AR chromatography system was employed, which includes a manual sample handling system (7725i), a binary pump, and a SPD-20 UV detector (Shimadzu International Trading Co., Ltd.).

### 2.2 Methods

#### 2.2.1 Preparation of the standard curve for luteolin

The standard curve and quantification of luteolin were established following the methodology described by Giacometti et al. [24]. The chromatographic system employed was a Shimadzu 2010AR model, equipped with a C18 Diamonsil analytical column (250 mm × 4.6 mm, 5 μm). The analysis was conducted using a SPD-20 UV detector, calibrated to a wavelength of 350 nm. The mobile phase was composed of a constant ratio elution, with components A and B in a 30:70 ratio. Component A was a 0.1 % solution of formic acid in water, and component B was acetonitrile. The flow rate was maintained at 1.0 mL/min, and the column temperature was precisely controlled at 30 °C. An injection volume of 20 μL was utilized.

To prepare the standard solution, accurately weigh out 10.0 m g of luteolin, dissolve it in methanol, and make up to 10 ml to obtain a stock solution of 1.0 mg/mL. Serial dilutions were created by transferring 0.1, 0.3, 0.5, 0.7, and 0.9 mL of the 1.0 mg/mL luteolin solution into separate 10 mL volumetric flasks, which were then filled to the mark with methanol. These solutions were thoroughly mixed before analysis. The standard curve for luteolin included concentrations ranging from 0.01 mg/mL to 0.09 mg/mL (R^2^ = 0.9995), with the calibration equation expressed as $y=7.0\times 10^{7}x-74091$, where x represents the sample concentration and y denotes the peak area.

#### 2.2.2 Extraction of luteolin from peanut shells

A precise weight of 0.5 g of peanut shell powder was placed in a round-bottom flask, and 2 mol/L ILs were added. The mixture underwent ultrasonic extraction, after which the resulting extract was centrifuged at 5000 rpm for 5 min to separate the solid residue. The supernatant is filtered through a 0.45 μm filter membrane prior to HPLC analysis. The luteolin extract was identified by comparing its retention time with that of the standard, and quantification was performed using the standard curve.

#### 2.2.3 Calculation of the luteolin content in peanut shells

The peak area was determined using the methodology outlined in section 2.2.1. The luteolin content in the peanut shell samples was calculated according to Equation (1):

(1) $$X=\frac{c\times V\times N}{M}$$

In this equation, *X* denotes the luteolin content in the peanut shell samples, expressed in mg/g; *c* represents the concentration calculated from the luteolin standard curve equation, in mg/mL; *V* is the volume of the luteolin extract from peanut shells, in mL; *N* is the dilution factor; and *M* is the mass of the peanut shell powder, in g.

#### 2.2.4 Screening of ILs

The extraction efficiency was assessed by screening seven different types of ILs. The ILs tested included 1-butyl-3-methylimidazolium chloride, 1-butyl-3- methylimidazolium bromide, 1-butyl-3-methylimidazolium tetrafluoroborate, 1-butyl- 3-methylimidazolium hexafluorophosphate, 1-butyl-3-methylimidazolium hydrogen sulfate, 1-ethyl-3-methylimidazolium chloride, and 1-ethyl-3-methylimidazolium bromide. The extraction process was conducted through ultrasonic treatment as detailed in section 2.2.2, utilizing the following parameters: ultrasonic power of 200 W, temperature of 60°C, extraction duration of 30 min, and a liquid–solid ratio of 20 mL/g. HPLC analysis was performed on the extracted samples, and the luteolin content was calculated using the standard curve and formula from 2.2.1, 2.2.3.

#### 2.2.5 Single factor experiment

A precise weight of 0.5 g of ground peanut shells was used to evaluate the effects of various parameters on the extraction yield of luteolin. The parameters under investigation included ultrasonic power levels (150 W, 175 W, 200 W, 225 W, and 250 W), extraction times (20 min, 30 min, 40 min, 50 min, and 60 min), extraction temperatures (40°C, 50°C, 60°C, 70°C, and 80°C), concentrations of IL (1.0 mol/L, 1.5 mol/L, 2.0 mol/L, 2.5 mol/L, and 3.0 mol/L), and liquid–solid ratios (10 mL/g, 20 mL/g, 30 mL/g, 40 mL/g, and 50 mL/g). Each factor was systematically varied while the others were held constant to elucidate their individual and collective influence on extraction efficiency.

#### 2.2.6 Optimization of UAE methods by RSM

Following the results of the single-factor experiments, the Box-Behnken design (BBD) method was employed to further optimize the ultrasonic power, extraction time, IL concentration, and liquid −solid ratio (Table 1). For each factor, three levels closest to the maximum extraction rate of luteolin were selected. In order to create a second-order polynomial model, a four-factor, three-level response surface analysis experiment was carried out with 29 random combinations. To find the best extraction conditions, the ideal values and matching experimental circumstances were filtered and forecasted.

**Table 1:** Coding of experimental parameters and related levels.

Experimental parameters	Unit	Coded values			
		Low (−1)	Medium (0)	High (+1)	
Ultrasonic power	W	X_1_	175	200	225
Extraction time	min	X_2_	40	50	60
Ionic liquid concentration	mol/L	X_3_	2	2.5	3.0
Liquid-solid ratio	mL/g	X_4_	30	40	50

#### 2.2.7 Optimization of UAE methods by artificial neural networks-genetic algorithm

The Back Propagation (BP) neural network is a type of artificial neural network (ANN) comprising an input layer, hidden layers, and an output layer [25]. Signals from the input layer are sequentially passed through each hidden layer before reaching the output layer, with each layer’s output affecting only the subsequent layer. ANNs possess robust machine learning capabilities and fault tolerance, enabling them to predict the error between calculated and measured values, adjust weights layer by layer, and iteratively train to minimize error.

In this study, an ANN was utilized for the experimental optimization of the luteolin extraction process. The 29 data sets obtained from the response surface experiment were used to train the ANN. The input layer consisted of four nodes representing ultrasonic power, extraction time, IL concentration, and liquid −solid ratio, while the output layer represented the extraction rate of luteolin. MATLAB R2019b software was employed as the neural network toolbox.

The model’s accuracy was enhanced through continuous training, tailored to determine the optimal number of nodes. The number of neurons in the input and output layers was established based on BBD data, making the number of hidden layer neurons a critical factor affecting model accuracy. Thus, it was essential to optimize the number of hidden layers. The data set was divided as follows: 70 % for training, 15 % for validation, and 15 % for testing. According to Kolmogorov’s theorem, the number of hidden layer neurons (n_2_) can be determined by the following equation (2):

(2) $$n_{2}⩽n_{1}+m+a$$

where n_1_ denotes the quantity of nodes in the input layer, m indicates the count of nodes in the output layer, and a be a constant that fluctuates between 1 and 10. Within this research, the quantity of nodes in the hidden layer exhibited variability, extending from 3.24 to 12.24. Testing networks with different numbers of nodes revealed that a high training accuracy was achieved with 9 hidden layer nodes. There was a learning rate of 0.01; a crossover chance of 0.7; a mutation probability of 0.1; and a maximum of 100 training repetitions.

#### 2.2.8 Interaction energy analysis

To elucidate the mechanism of ILs-UAE of luteolin, interactions between ILs and luteolin molecules during the extraction process were simulated. The molecular structures of the ILs and luteolin were constructed using Materials Studio software. Subsequently, these structures were geometrically optimized using the fastest descent method and the conjugate gradient method within the Forcite module to obtain the lowest-energy geometric configurations. The electrostatic interaction energy, van der Waals forces, and total energy were computed using a Gromacs-based method to determine the interaction energy between luteolin and ILs. The interaction energy between the two molecules can be calculated using the following equation (3):

(3) Eint=Ecluster-(EA+EB)

where *E_int_* is the interaction energy, *E_cluster_* is the total energy of the molecular cluster composed of luteolin and ILs, and *E_A_* and *E_B_* are the energies of molecules A and B, respectively.

#### 2.2.9 Extraction dynamic simulation

Following the method described by Wang et al. [26], three dynamic models with high fitting degrees—the first-order rate model, the second-order rate model, and Fick’s second law model—were selected to perform regression analysis on the changes in luteolin yield from peanut shells. This analysis was conducted at an ultrasonic temperature of 70°C and over an ultrasonic time range of 20 to 60 min. The dynamic equations for luteolin extraction were established, allowing for the determination of the optimal kinetic model for luteolin extraction through comparative analysis.

#### 2.2.10 Comparison with traditional methods and scanning electron microscopy (SEM) analysis

In this research, a comparative evaluation was performed among three distinct extraction techniques, along with the ILs-UAE method. Pure water served as the reference solvent for extracting luteolin from peanut shells using ILs-UAE. Extraction experiments were performed under optimized conditions, with the type of solvent as the only variable. Specifically, 20 mL of clean water and 0.5 g of dried plant material were combined, and the mixture was ultrasonically treated for 50 min at a power of 200 W and an extraction temperature of 70 °C. The peak area was determined using HPLC according to the method described in section 2.2.1, and the luteolin content was determined using the standard curve. Then, using the above formula, the luteolin content was calculated.

For the hot reflux extraction, the solvent of choice was a 70 % ethanol solution. 30 mL of a 70 % ethanol solution was used to reflux weighed samples (1 g) for two hours. After filtering and diluting the extract to the necessary concentration, the luteolin content was determined using the standard curve and formula described in section 2.2.1, in accordance with the ultrasonic water technique.

Scanning electron microscopy (SEM) with a tungsten wire probe was used to examine surface microstructural changes of several treated powder samples at an acceleration voltage of 18 kV. A) samples that were air-dried subsequent to being crushed; B) an air-dried sample was extracted using ultrasonic treatment with pure water; C) an air-dried sample that underwent hot reflux extraction with a 70 % ethanol solution; and D) an air-dried sample that received treatment with ionic liquids. Several measurements were made at magnifications between 150× and 1000×.

### 2.3 Statistical analysis

Statistical analysis of the experimental data was conducted using Design-Expert 13.0, MATLAB R2019b, and SPSS 24.0. All results are presented as mean ± standard deviation (SD), derived from a minimum of three independent experiments (n = 3), with each experiment comprising a minimum of three replicates per sample. A one-way analysis of variance (ANOVA), followed by a Tukey's post hoc test, was employed to compare the samples, with the significance threshold set at p < 0.05. Regression equations for the evaluated responses were derived, and the contribution and significance of each parameter were assessed. Additionally, response surface plots were generated to determine optimal and suboptimal conditions for extraction, employing RSM through Design Expert. Furthermore, MATLAB was utilized for constructing, testing, and validating the ANN.

## 3 Results and discussion

### 3.1 Screening of the anions

**Fig. 1:**
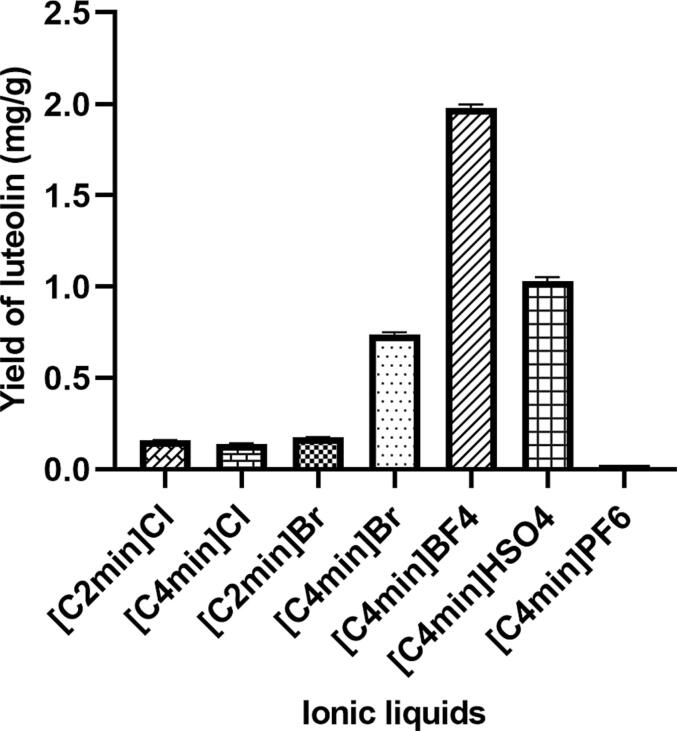
The effect of ionic liquid types on the extraction rate of luteolin from peanut shells.

Seven ILs were evaluated for their effectiveness in luteolin extraction from peanut shells in order to examine the impact of various ILs on the extraction rate of luteolin. As shown in Fig. 1, different kinds of ILs had a major impact on the luteolin extraction rate. Notably, the extraction utilizing 1-butyl-3-methylimidazolium tetrafluoroborate (BF_4_^-^) yielded the highest content, reaching 1.98 ± 0.01 mg/g. In contrast, the extraction rate using1-butyl-3-methylimidazolium hexafluorophosphate (PF_6_^-^) was considerably lower, at only 0.02 ± 0.01 mg/g.

These findings indicate that the extraction rate of luteolin increased progressively with the use of bromide (Br^−^) as the anion and as the alkyl substitution chain length of the imidazolium cation extended from C2 to C4. This trend could be linked to the intensification of van der Waals interactions between luteolin and the ILs as the alkyl chain length extends, consequently improving the extraction efficiency. When using [C_4_min]^+^ as the cation, ILs containing BF_4_^-^ exhibited superior extraction efficiency for luteolin compared to those with Br^-^, Cl^-^, HSO_4_^-^, and PF_6_^-^. This superiority can be attributed to BF_4_^-^ being a relatively large anion with high electron cloud density, facilitating the formation of robust ion-pair interactions with [C_4_min]^+^. Such strong interactions significantly improve the dissolution and extraction efficiency of luteolin from plant materials. Conversely, Br^-^, Cl^-^, HSO_4_^-^, and PF_6_^-^ exhibited weaker interactions with [C_4_min]^+^, resulting in lower extraction efficiencies [27].

### 3.2 Single-factor test

#### 3.2.1 Influence of ultrasonic power on the extraction rate of luteolin

**Fig. 2:**
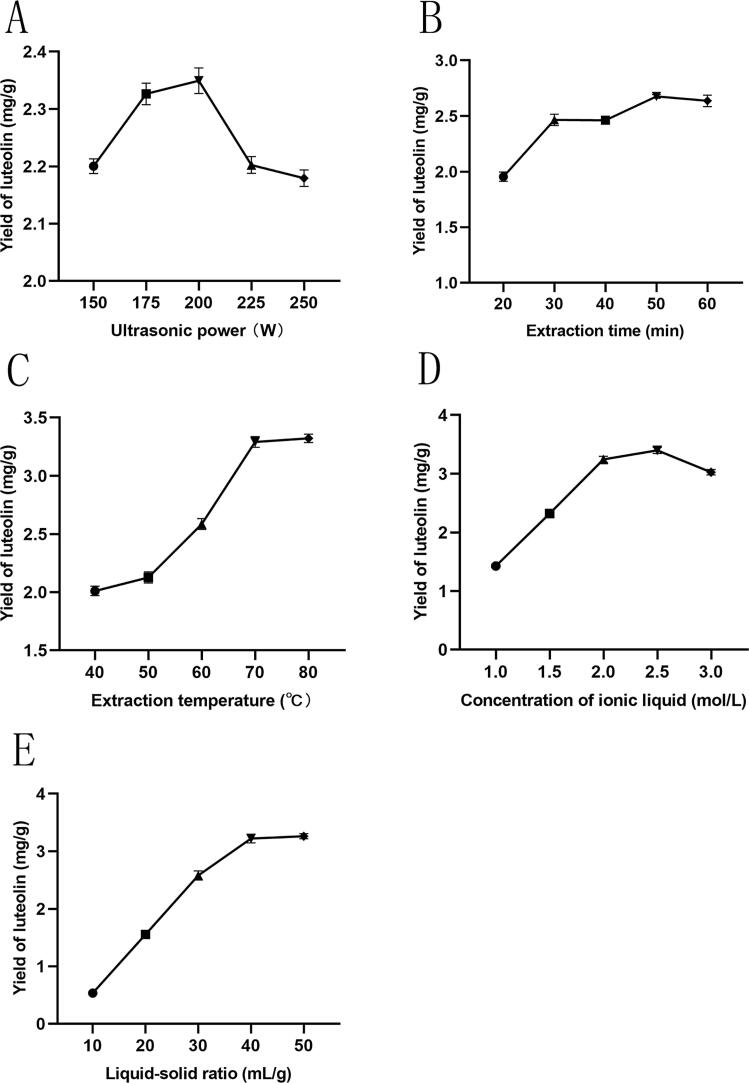
The influence of different factors on the extraction rate of luteolin from peanut shells. (A) Ultrasound power, (B) extraction time, (C) extraction temperature, (D) ionic liquid concentration, (E) liquid–solid ratio.

The influence of ultrasonic power on the yield of luteolin is depicted in Fig. 2A. As ultrasonic power increased, the extraction rate of luteolin initially rose before subsequently declining, with a peak at 200 W, achieving a value of 3.35 ± 0.05 mg/g. Beyond this point, further increases in ultrasonic power resulted in a decrease in the extraction rate. This behavior can be explained by the increasing breakdown of raw material cells brought on by the escalating impacts of ultrasonic cavitation, mechanical action, and thermal effects. These factors facilitate the continuous release of intracellular substances, enhancing solute diffusion and promoting a more complete dissolution of luteolin in the ILs, thereby improving the extraction rate [28]. However, excessively high ultrasonic power may lead to the degradation of the released flavonoids, ultimately reducing the extraction rate of luteolin. Additionally, it may encourage the release of other impurities, thereby diminishing the purity of the luteolin in the extract. This leads to the conclusion that 200 W is the ideal ultrasonic power.

#### 3.2.2 Influence of extraction time on the extraction rate of luteolin

The extraction of active components from natural products requires a specific amount of time to achieve equilibrium in the extraction process. Fig. 2B illustrates that the extraction efficiency of luteolin rises progressively as the duration of ultrasonic treatment is extended. The highest extraction rate of luteolin (2.66 ± 0.03 mg/g) was observed at 50 min, after which the extraction rate began to decline gradually with further extension of ultrasonic time.

In the initial phase, the short ultrasonic duration likely inhibited the breakdown of peanut shell cells by the ultrasonic mechanical energy, limiting the ability of the extraction solvent to penetrate and interface effectively with the active components, resulting in insufficient extraction. As the ultrasonic time increased, complete penetration of the peanut shell cells occurred, thereby enhancing the extraction rate of luteolin. However, prolonged ultrasonic exposure can lead to the dissolution of various other components, which may cause thermal instability and degradation of luteolin, ultimately diminishing its extraction rate [29]. The ideal extraction time for ultrasonic application was determined to be 50 min based on this research.

#### 3.2.3 Influence of extraction temperature on the extraction rate of luteolin

The temperature during UAE has a major impact on the rate at which luteolin is extracted from peanut shells, as illustrated in Fig. 2C. As the temperature rises, the movement of intracellular molecules and the speed of free diffusion are accelerated, facilitating the transfer of cell contents to the solvent, allowing the ILs to more effectively extract luteolin from the sample. At temperatures of 70°C (3.29 ± 0.05 mg/g) and 80°C (3.32 ± 0.02 mg/g), the increase in extraction rate diminished, with both temperatures yielding comparable extraction rates. Therefore, when the extraction time is set at 50 min and the ultrasonic power at 200 W, higher temperatures are associated with higher extraction rates. However, considering the stability and energy efficiency of luteolin, an extraction temperature of 70°C is deemed optimal.

#### 3.2.4 Influence of IL concentration on the extraction rate of luteolin

The concentration of IL is a primary factor influencing the extraction rate of active plant components. Under the conditions outlined in Fig. 2D, it is evident that the extraction efficiency of luteolin exhibited a consistent rise as the concentration of IL escalated, specifically within the range of 1.0 to 2.5 mol/L. At the 2.5 mol/L concentration, the extraction efficiency of luteolin peaked, attaining a maximum value of 3.36 ± 0.05 mg/g. However, an incremental rise in concentration from 2.5 to 3.0 mol/L resulted in a marginal decline in extraction efficiency. This phenomenon could be explained by the significant effects of IL concentration on cell wall swelling and disruption as well as the extraction solvent's mass transfer process, which directly affects the target compound's extraction efficiency. An appropriate concentration of IL can enhance the degradation of plant cell walls. However, when the IL concentration exceeds a certain threshold, the resulting increase in viscosity impairs the diffusion capability of the ILs, making it difficult for them to penetrate the tissues of the medicinal materials and adequately extract the target compounds from traditional Chinese herbs [30]. Thus, a concentration of 2.5 mol/L of IL is considered optimal.

#### 3.2.5 Influence of liquid-solid ratio on the extraction rate of luteolin

Since the liquid–solid ratio affects the area of contact between the solvent and the raw material, it is a crucial component in luteolin extraction efficiency. As depicted in Fig. 2E, the extraction efficiency of luteolin initially ascended and then gradually plateaued with an increasing liquid–solid ratio. The extraction rate of luteolin increased quickly between 10 and 40 mL/g, peaking at 3.30 ± 0.07 mg/g at a liquid–solid ratio of 40 mL/g. Beyond this point, when the liquid–solid ratio exceeded 40 mL/g, the extraction rate of luteolin did not show a significant increase.

This trend may be attributed to the enhanced interaction between peanut shell powder and the solvent, which facilitates the solubility of luteolin in the extract. However, at excessively high liquid–solid ratio, the dissolution of luteolin from the peanut shell into the ILs becomes saturated, leading to negligible changes in the extraction rate [31]. Consequently, the optimal extraction rate of luteolin is achieved at a liquid–solid ratio of 40 mL/g.

### 3.3 Optimization of the extraction of luteolin using ionic liquids via RSM

#### 3.3.1 Model fitting and statistical analysis

Single-factor experiments can only reflect the influence of one variable on the extraction rate; therefore, it is essential to consider the interactions among multiple influencing factors when designing multi-factor experiments. RSM optimization generally outperforms traditional single-factor optimization in extracting natural compounds, owing to the interactions among different variables. Based on the findings from the single-factor tests, four factors were selected for optimization following the Box-Behnken design principle: ultrasonic power, extraction time, IL concentration, and liquid–solid ratio. Utilizing Design-Expert 13.0 software, a response surface design featuring four-factor and three-level was established. This resulted in 29 experimental sets based on the Box-Behnken response surface design, with five sets of repeated tests conducted at the central point to estimate the experimental error. The outcomes of these experiments are summarized in Table 2. Design-Expert 13.0 software facilitated the binomial fitting of the data from Table 2, followed by variance analysis of the model. The derived binomial fitting equation is as follows (4):

(4) $$Y=3.62+0.0417X_{1}+0.0373X_{2}+0.1126X_{3}+0.0831X_{4}-0.0735X_{1}X_{2}+0.1112X_{1}X_{3}-0.0222X_{1}X_{4}+0.1505X_{2}X_{3}+0.0770X_{2}X_{4}+0.0610X_{3}X_{4}-0.1587X_{1}^{2}-0.1800X_{2}^{2}-0.1516X_{3}^{2}-0.1566X_{4}^{2}$$

**Table 2:** List of experimental values and predicted values from RSM and ANN.

Run	Independent variables				The yield of luteolin (mg/g)		
	Ultrasonic power (W)	Extraction time (min)	Ionic liquid concentration (mol/L)	Liquid-solid ratio (mL/g)	Actual values	RSM	ANN
						predicted	predicted
1	175	40	2.5	40	3.10	3.13	3.59
2	225	40	2.5	40	3.32	3.36	3.59
3	175	60	2.5	40	3.40	3.35	3.59
4	225	60	2.5	40	3.32	3.29	3.58
5	200	50	2	30	3.22	3.18	3.10
6	200	50	3	30	3.31	3.28	3.32
7	200	50	2	50	3.20	3.22	3.40
8	200	50	3	50	3.53	3.57	3.32
9	175	50	2.5	30	3.09	3.16	3.22
10	225	50	2.5	30	3.23	3.29	3.30
11	175	50	2.5	50	3.44	3.37	3.20
12	225	50	2.5	50	3.48	3.41	3.53
13	200	40	2	40	3.31	3.29	3.09
14	200	60	2	40	3.06	3.06	3.23
15	200	40	3	40	3.23	3.22	3.44
16	200	60	3	40	3.58	3.59	3.48
17	175	50	2	40	3.25	3.27	3.31
18	225	50	2	40	3.12	3.13	3.06
19	175	50	3	40	3.27	3.27	3.42
20	225	50	3	40	3.59	3.58	3.32
21	200	40	2.5	30	3.30	3.24	3.25
22	200	60	2.5	30	3.18	3.16	3.12
23	200	40	2.5	50	3.23	3.25	3.27
24	200	60	2.5	50	3.42	3.48	3.59
25	200	50	2.5	40	3.52	3.62	3.30
26	200	50	2.5	40	3.69	3.62	3.18
27	200	50	2.5	40	3.7	3.62	3.24
28	200	50	2.5	40	3.61	3.62	3.42
29	200	50	2.5	40	3.59	3.62	3.59

The variance analysis of the regression model obtained in this study is summarized in Table 3. The *F*-value for the regression model is 15.21, with a significance (*p* < 0.0001). This indicates that the established model for the luteolin extraction rate is highly significant and reliable. The main order of influence of factors on the extraction rate of luteolin, based on the *F*-value, is as follows: (*X_3_* > *X_4_* > *X_1_* > *X_2_*), indicating that IL concentration (*X_3_*) has the greatest impact, followed by liquid–solid ratio (*X_4_*), ultrasonic power (*X_1_*), and extraction time (*X_2_*). The *p*-value for the lack of fit term is 0.7398 (greater than 0.05), suggesting that this term is not significant and reinforces the regression equation's adequacy. Furthermore, the adjusted coefficient of determination (R^2^) is 0.9383, implying a good fit between the regression model and actual data, with a small experimental error. The adjusted correlation coefficient (R^2^_adj_) is also 0.8766, indicating that 87.66 % of the variability in luteolin yield can be explained by the selected variables.

**Table 3:** ANOVA for Quadratic model.

Source	Sum of Squares	df	Mean Square	F-value	p-value	
Model	0.9115	14	0.0651	15.21	< 0.0001	Significant
X_1_-ultrasonic power	0.0208	1	0.0208	4.87	0.0446	
X_2_- Extraction time	0.0167	1	0.0167	3.91	0.0681	
X_3_- IL concentration	0.1521	1	0.1521	35.53	< 0.0001	
X_4_- liquid–solid ratio	0.0828	1	0.0828	19.35	0.0006	
X_1_X_2_	0.0216	1	0.0216	5.05	0.0413	
X_1_X_3_	0.0495	1	0.0495	11.56	0.0043	
X_1_X_4_	0.0020	1	0.002	0.4626	0.5075	
X_2_X_3_	0.0906	1	0.0906	21.16	0.0004	
X_2_X_4_	0.0237	1	0.0237	5.54	0.0337	
X_3_X_4_	0.0149	1	0.0149	3.48	0.0833	
X_1_2	0.1634	1	0.1634	38.18	< 0.0001	
X_2_2	0.2101	1	0.2101	49.09	< 0.0001	
X_3_2	0.1491	1	0.1491	34.83	< 0.0001	
X_4_2	0.1591	1	0.1591	37.16	< 0.0001	
Residual	0.0599	14	0.0043			
Lack of Fit	0.037	10	0.0037	0.6436	0.7398	Not significant
Pure Error	0.023	4	0.0057			
Cor Total	0.9714	28				

The coefficient of variation (C.V.%) is 1.95 %, which demonstrates strong model fit and its applicability for prediction and analysis. Notably, the primary terms (*X_3_*) and (*X_4_*) in the regression model are highly significant to the luteolin extraction rate, while the interaction terms (*X_1_X_3_*) and (*X_2_X_3_*) are also significant. Additionally, the interaction terms (*X_1_X_2_*) and (*X_2_X_4_*) exhibit significant effects on the extraction rate, with extreme significance observed in the quadratic terms (*X_1_^2^*), (*X_2_^2^*), (*X_3_^2^*), and (*X_4_^2^*). In conclusion, the established model demonstrates high accuracy and reliability, making it suitable for the analysis and prediction of luteolin extraction.

#### 3.3.2 RSM analysis

**Fig. 3:**
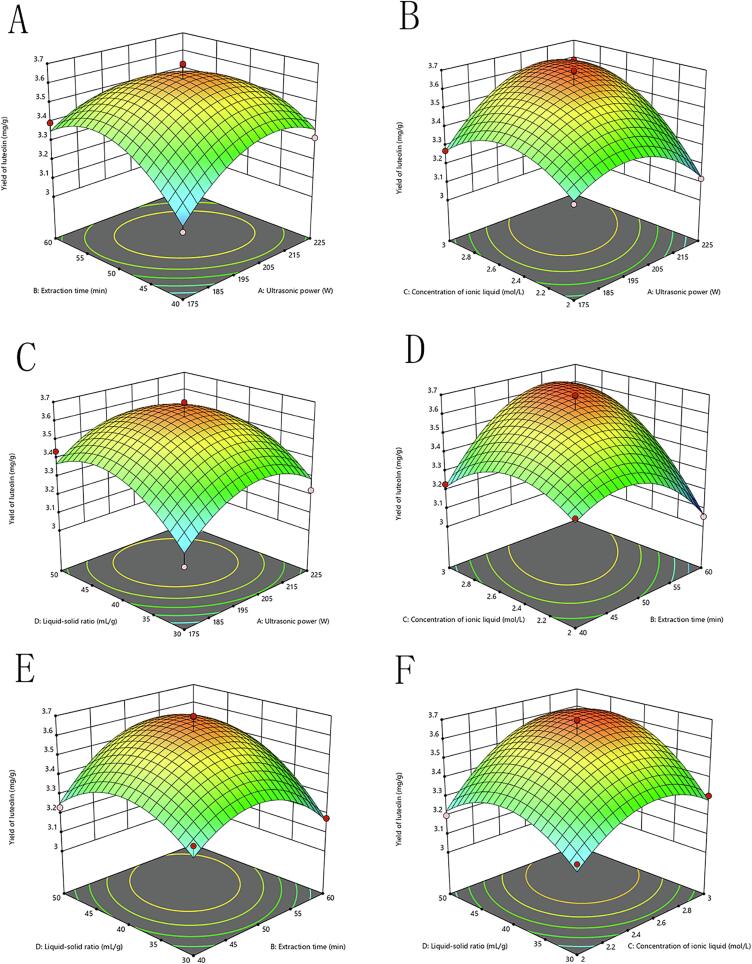
3D curve graphs of the extraction rate of luteolin from peanut shells under different extraction parameters. (A) Ultrasonic power and extraction time, (B) Ultrasonic power and ionic liquid concentration, (C) Ultrasonic power and solid-to-liquid ratio, (D) Extraction time and ionic liquid concentration, (E) Extraction time and solid-to-liquid ratio, (F) Ionic liquid concentration and solid-to-liquid ratio.

**Fig. 4:**
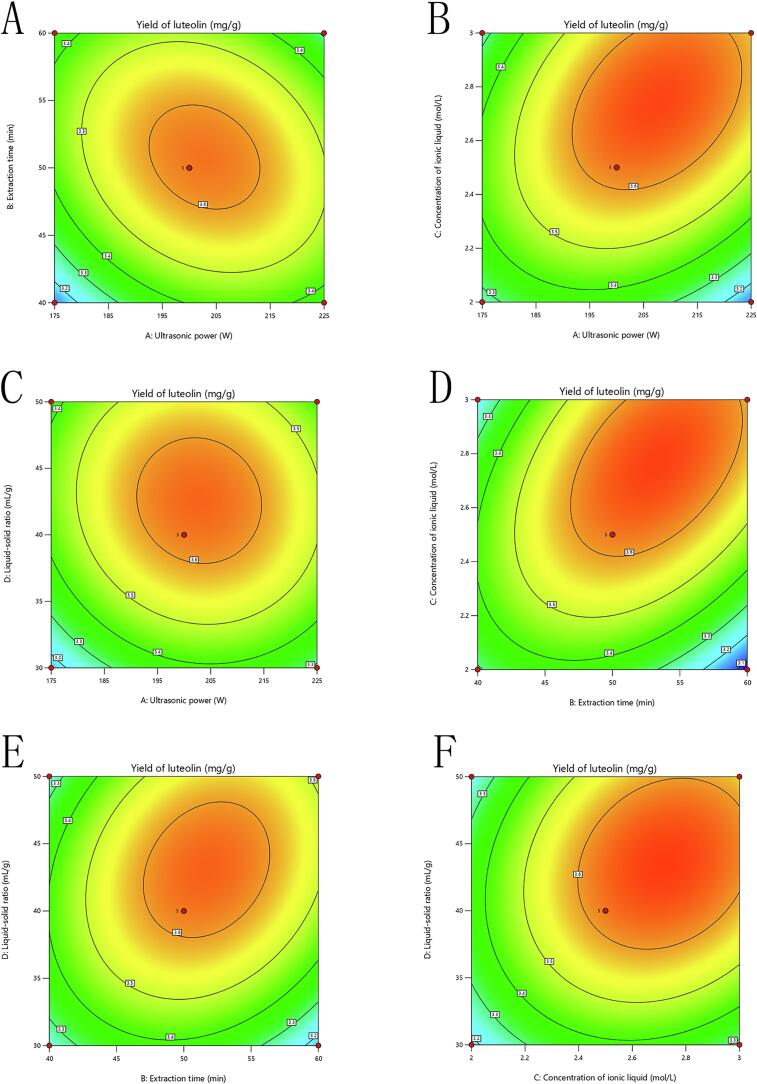
Contour maps of the extraction rate of luteolin from peanut shells under different extraction parameters. (A) Ultrasonic power and extraction time, (B) Ultrasonic power and ionic liquid concentration, (C) Ultrasonic power and solid-to-liquid ratio, (D) Extraction time and ionic liquid concentration, (E) Extraction time and solid-to-liquid ratio, (F) Ionic liquid concentration and solid-to-liquid ratio.

The response surface map not only illustrates the influencing factors on sensory scores but also highlights the interactions between these factors. The degree of influence of each factor on the response value is depicted in the response surface diagram; a steeper 3D surface indicates a more significant impact of the factor. Additionally, contour plots provide insight into the significance of the interaction between two factors; a contour shape closer to an ellipse indicates stronger significance. In contrast, a flat surface with sparse and nearly circular contours suggests minimal influence of the variables on the response value and a lack of obvious interaction. As presented in Fig. 3, Fig. 4, the interactions of *X_1_X_3_* and *X_2_X_3_* have a very significant effect on luteolin, manifested as steep slopes and more elliptical contours in the three-dimensional maps (Fig. 3B-D; 4B-D). Conversely, the interactions of *X_1_X_4_* and *X_3_X_4_* exhibit no significant influence on luteolin, as indicated by the nearly circular contours (Fig. 3C-F; 4C-F). These findings align with the data presented in Table 3.

### 3.4 Analysis of conditions for optimizing luteolin yield by ANN-GA

**Fig. 5:**
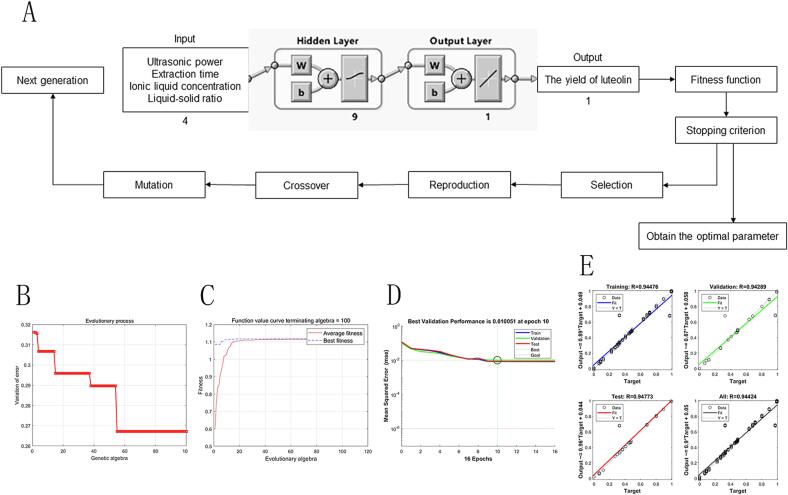
(A) the optimal architecture of developing the ANN-GA model, (B) the error variation of the ANN-GA model with different generations, (C) the Fitness curve with evolutionary algebra, (D) network training curves with Epochs number for trained subsets, (E) regression coefficient of experimental data and ANN-GA model.

Based on the RSM, a neural network was trained and simulated using ANN-GA to model the relationship between the output variables and the four inputs. The model-building process included data collection, network construction, configuration, weight and bias initialization, data training, testing, and subsequent validation. MATLAB R2019b software was utilized for programming, resulting in a 4–9-1 neural network model for testing and predictions (Fig. 5A). Initially, the error was approximately 0.318, representing the algorithm's starting state or the error level of a random solution. As genetic iterations progressed, the error gradually decreased; notably, after the 40th generation, there was a sharp drop in error variation, suggesting that the algorithm identified a more efficient solution or entered a better search space. Eventually, around the 60th generation, the error variation reached its lowest point and stabilized at approximately 0.27, indicating that the algorithm had achieved a favorable equilibrium point, with no significant improvements in subsequent iterations (Fig. 5B-C). In terms of MSE parameters, performance evaluations demonstrate that the network's performance improves with the number of epochs during training, validation, and testing. The best validation performance occurs with an MSE value of 0.0101 after 10 epochs (Fig. 5D). The R-value of the fitting regression coefficient of the model trained by BP reflects the correlation between the target value and the output value. To further evaluate the performance of the training model, scatter plots of the training, validation, testing, and prediction sets were generated. As illustrated in Fig. 5E, the fitted curve closely aligns with Y = X, indicating strong compatibility between the test values and the predicted values. The correlation coefficients for the training regression curve (R = 0.9447), the verification regression curve (R = 0.9429), the testing regression curve (R = 0.9477), and the overall regression curve (R = 0.9442) confirm the model's robustness.

### 3.5 RSM and ANN-GA optimization results and test verification

Two separate techniques, RSM and ANN-GA, were utilized to optimize the extraction of luteolin. As summarized in Table 4, the ideal parameters identified by RSM were 206 W of ultrasonic power, 55.7 min of extraction duration, 2.8 mol/L of IL concentration, and 45.7 mL/g of liquid–solid ratio. In comparison, the optimal parameters identified through ANN-GA were 205.7 W for ultrasonic power, 52.4 min for extraction time, 2.6 mol/L for IL concentration, and 44.2 mL/g for the liquid–solid ratio. To validate the extraction process, three trials were conducted under the conditions established by both RSM and ANN-GA optimization. The data analysis presented in Table 4 indicates that RSM outperformed the ANN-GA model concerning the optimization of luteolin yield, achieving a relative error of only 0.27 % compared to1.05 % for ANN-GA. Moreover, the yield of luteolin optimized by RSM reached 3.71 ± 0.06 mg/g, surpassing the 3.57 ± 0.02 mg/g yield obtained through ANN-GA optimization.

**Table 4:** Predicted and experimental values of the responses at optimum conditions.

	Optimum condition					The yield of luteolin (mg/g)		Relative error (%)
	Ultrasonic power (W)	Extraction time (min)	Ionic liquid concentration (mol/L)	liquid–solid ratio (mL/g)		actual value	predicted value	
RSM ANN	206.0 205.7	55.7 52.4	2.8 2.6	45.7 44.2		3.71 3.57	3.70 3.65	0.27 2.24

These results demonstrate that the RSM model exhibits greater accuracy in fitting, estimation, and prediction, yielding superior optimization results. The simplified structure and interpretability of the RSM model may enhance its effectiveness for such optimization tasks, particularly when data availability is limited, but it may be constrained when dealing with complex systems. In contrast, ANN-GA has strong capabilities in handling complex data, with the advantage of global search, but it has larger errors, higher data requirements, and poorer stability. In summary, RSM has advantages in polynomial model optimization and small sample data processing, while ANN-GA is theoretically suitable for more complex relationships. Consequently, the findings of this experiment affirm the applicability and reliability of the RSM method in optimizing luteolin yield, highlighting the feasibility of the approach and the credibility of the experimental results.

### 3.6 Analysis of intermolecular interaction energy

**Fig. 6:**
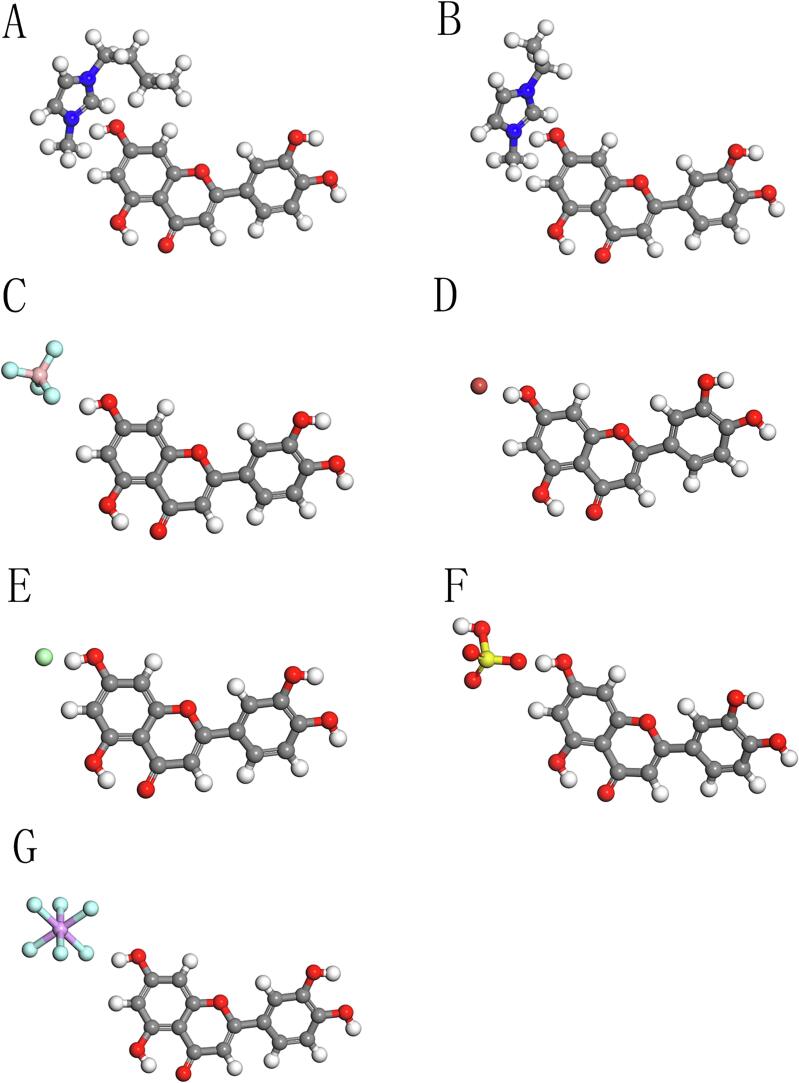
Molecular structures of the interaction between luteolin and ionic liquids. (A) Luteolin with [C_4_ mim]^+^ , (B) Luteolin with [C_2_ mim]^+^ , (C) Luteolin with [BF_4_ ]^-^ , (D) Luteolin with Br^-^ , (E) Luteolin with Cl^-^ , (F) Luteolin with [HSO_4_ ]^-^ , (G) Luteolin with [PF_6_ ]^-^ .

In this study, Materials Studio software was employed to optimize the structures of luteolin and ILs, and the interaction energy between them was analyzed. The findings are illustrated in Fig. 6. The structures of luteolin and the imidazole cations, [C_2_mim]^+^ and [C_4_mim]^+^, are depicted in Fig. 6A and 6B, respectively. The imidazole ring is characterized as a five-membered aromatic heterocyclic structure containing two nitrogen atoms, where the hydrogen atom attached to one nitrogen atom can easily dissociate, resulting in a positively charged imidazole ring. This charge facilitates the formation of hydrogen bonds with hydroxyl groups present in the luteolin structure, as well as other intermolecular interactions.

The interaction energy calculated between luteolin and the cation [C_4_mim]^+^ is − 23.85 kJ/mol, which is greater in magnitude (more negative) than that between luteolin and [C_2_mim]^+^ (−18.02 kJ/mol). This suggests a stronger binding affinity between luteolin and [C_4_mim]^+^, implying that ionic liquids containing [C_4_mim]^+^ may exhibit superior extraction efficiency for luteolin compared to those containing [C_2_mim]^+^.

Regarding the anions examined—[BF_4_]^−^, [Br]^−^, [Cl]^−^, [HSO_4_]^−^, and [PF_6_]^−^—the interaction energies with luteolin were − 35.92 kJ/mol, −17.57 kJ/mol, −12.78 kJ/mol, −19.30 kJ/mol, and − 10.92 kJ/mol, respectively (as shown in Fig. 6C-G). The comparatively stronger interaction energy observed for [BF_4_]^−^ can be attributed to the highly electronegative fluorine atoms, which are capable of forming strong hydrogen bonds with hydroxyl or other hydrogen-containing groups within the luteolin molecule [32]. Conversely, [PF_6_]^−^ displayed the weakest interaction energy with luteolin, which may be due to the large steric hindrance presented by its fluorine atoms. Despite being electronegative, these fluorine atoms do not easily engage in the formation of effective hydrogen bonds with the hydroxyl or other functional groups present in the luteolin structure.

The above analysis indicates that [C_4_mim]BF_4_ possesses the most favorable extraction properties for luteolin, aligning with the experimental results obtained in this study. This suggests that the optimized interaction energies provide a robust basis for understanding and predicting the extraction efficiencies of different ionic liquids for luteolin extraction.

### 3.7 Extraction kinetics analysis

**Fig. 7:**
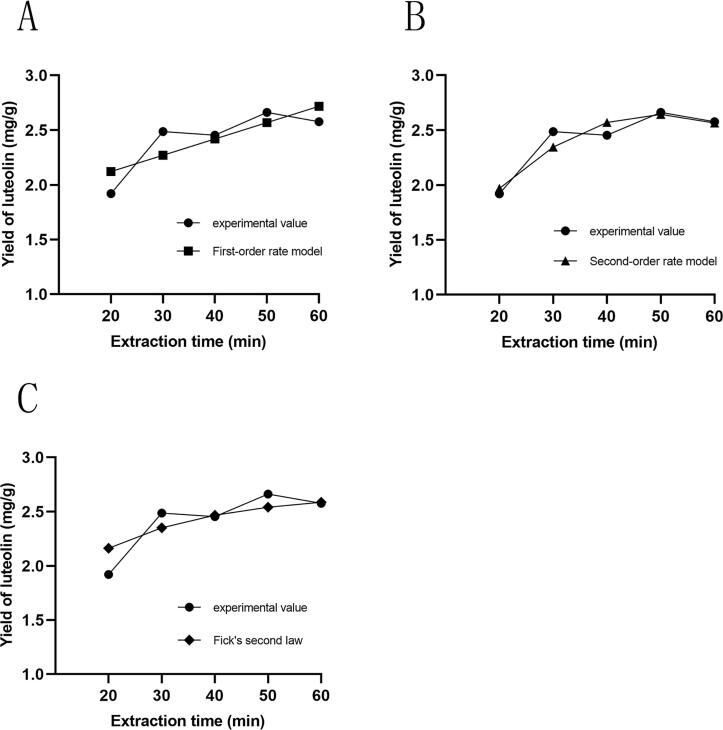
Analysis of different dynamic models. (A) First-order rate model, (B) Second-order rate model, (C) Fick's second law model.

In the study of UAE of luteolin, establishing a kinetic model is a crucial step for understanding the extraction process and optimizing operational conditions. By analyzing experimental data and employing mathematical modeling, this investigation utilized first-order, second-order, and Fick's second-law models to fit the parameters under optimal extraction conditions, thereby exploring the influence of ultrasonic time on the extraction rate. The results are presented in Table 5 and Fig. 7.

**Table 5:** Extraction kinetic model.

Model	Equation	R^2^
First-order rate model	y = 0.01491x + 1.823	0.6559
Second-order rate model	y = -0.0007579x2 + 0.07554x + 0.7618	0.8952
Fick’s second law model	y = 0.0474x + 0.7251	0.7955

As shown in Table 5, the coefficient of determination (R^2^) for the second-order rate model is 0.8952, indicating that the trend predicted by this model aligns closely with the observed experimental data (Fig. 7B). This suggests that the second-order rate model effectively reflects changes in the experimental data and accurately describes the extraction process of luteolin. In contrast, the R2 value for Fick's second law model is 0.7955. Although the trend predicted by this model is generally consistent with the experimental data, there are discrepancies in the specific values (Fig. 7C), indicating that while it can fit the experimental data reasonably well, it is not as reliable as the second-order model. The R^2^ for the first-order rate model is notably lower at 0.6559, suggesting an average fit to the experimental data (Fig. 7A). In summary, the extraction kinetics of luteolin are best represented by the second-order rate model.

### 3.8 Comparison of traditional extraction methods and SEM analysis

This study compared the effects of UAE using pure water with reflux extraction (HRE) utilizing 70 % ethanol, as well as with ILs-UAE. The yield of luteolin extracted with pure water (0.81 ± 0.15 mg/g) was significantly lower than that obtained with ILs-UAE (3.67 ± 0.05 mg/g), indicating that ILs play a crucial role in the extraction process. When comparing with the extraction efficiency of HRE using 70 % ethanol (2.35 ± 0.18 mg/g), ILs-UAE not only significantly reduced the extraction time but also markedly improved extraction efficiency.

**Fig. 8:**
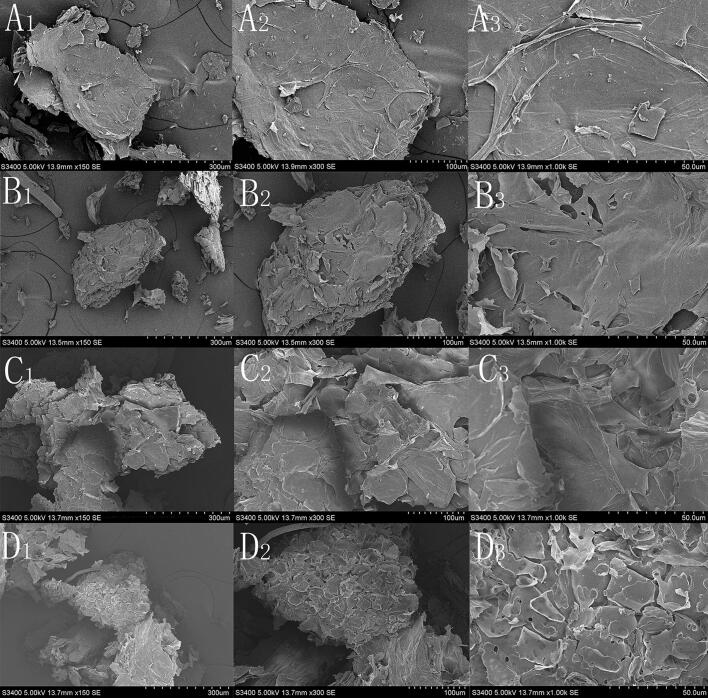
Comparison of Scanning Electron Microscopy of Luteolin Extracted by Different Methods. Untreated powder (A_1_ : ×150; A_2_ :× 300; A_3_ : ×1000), powder after reflux extraction with 70 % ethanol (B_1_ : ×150; B_2_ : × 300; B_3_ : ×1000), powder after ultrasonic water treatment (C_1_ : × 150； C_2_ : × 300; C_3_ : × 1000), and powder after ultrasonic-assisted ionic liquid extraction (D_1_ : × 150; D_2_ : × 300; D_3_ : × 1000).

To explore the extraction mechanism, microstructural changes in the samples were observed using SEM. Samples treated with four different extraction methods were placed on the SEM sample holder to analyze the surface morphology of the medicinal materials processed through various techniques, as illustrated in Fig. 8. The tissue structure of the peanut shell sample remained largely intact after grinding, with a relatively close arrangement, indicating minimal damage from the grinding treatment (Fig. 8A1-8A3). In comparison, the surface of the material subjected to 70 % ethanol reflux treatment appeared slightly wrinkled, yet its overall spatial structure was not significantly compromised (Fig. 8B1-8B3). In contrast, samples treated with ultrasonic water exhibited damage to the cell wall and cell tissue due to the cavitation effect of ultrasound, leading to cellular rupture and the formation of hollow structures (Fig. 8C1-8C3). The images from ILs-UAE revealed even greater tissue damage and more pronounced cavity structures (Fig. 8D1-8D3), indicating that the combined action of ILs and UAE results in increased fragmentation of plant cells. These data underline that the combination of ILs and UAE efficiently affects the microstructure of plant tissue, enabling solvent penetration and disintegration.

## 4 Conclusion

This study presents an UAE method for isolating luteolin from peanut shells, utilizing ILs as the extraction solvent. Following initial single-factor experiments, RSM and ANN-GA were employed for optimization. The results from RSM demonstrated greater accuracy compared to those obtained from ANN-GA. The optimal extraction conditions for luteolin were identified as an ultrasonic power of 206 W, an extraction time of 55.7 min, an IL concentration of 2.8 mol/L, and a liquid–solid ratio of 45.7 mL/g. Under these conditions, the highest luteolin yield achieved was 3.71 ± 0.06 mg/g. The interaction energy analysis revealed [C4mim]BF4 as the most effective IL for luteolin extraction. A second-order rate model accurately described the extraction kinetics. The integration of ILs with UAE not only improves extraction efficiency but also offers a greener, energy-efficient alternative to traditional methods, significantly reducing energy consumption and organic solvent use. This research presents a significant advancement in sustainable luteolin extraction technology.

## CRediT authorship contribution statement

**Liwei Niu:** Writing – original draft, Investigation. **Siwen Zhang:** Methodology, Investigation. **Xiaoyu Si:** Data curation. **Yuhan Fang:** Software. **Shuang Wang:** Validation. **Lulu Li:** Validation. **Zunlai Sheng:** Writing – review & editing, Conceptualization.

## Declaration of competing interest

The authors declare that they have no known competing financial interests or personal relationships that could have appeared to influence the work reported in this paper.

## References

[1] Liao X., Xie H.X., Hu Z.C., Wang J.N., Liu M.J., An J.Y., Wei H., Zhang H.J. (2024). Peanut-shelling technologies and equipment: a review of recent developments. Agriculture-Basel.

[2] Meng W.B., Shi J.Y., Zhang X.Y., Lian H., Wang Q.G., Peng Y. (2020). Effects of peanut shell and skin extracts on the antioxidant ability, physical and structure properties of starch-chitosan active packaging films. Int. J. Biol. Macromol..

[3] Imran A., Humiyion M., Arshad M.U., Saeed F., Arshad M.S., Afzaal M., Imran M., Usman I., Ikram A., Naeem U., Hussain M., Al Jbawi E. (2022). Extraction, amino acid estimation, and characterization of bioactive constituents from peanut shell through eco-innovative techniques for food application. Int. J. Food Prop..

[4] Ou H.C., Pandey S., Hung M.Y., Huang S.H., Hsu P.T., Day C.H., Pai P.Y., Viswanadha V.P., Kuo W.W., Huang C.Y. (2019). Luteolin: a natural flavonoid enhances the survival of HUVECs against oxidative stress by modulating AMPK/PKC pathway. Am. J. Chinese Med..

[5] Guo Y.R., Liu Y., Zhang Z.H., Chen M.H., Zhang D.X., Tian C.L., Liu M.C., Jiang G.T. (2020). The antibacterial activity and mechanism of action of luteolin against trueperella pyogenes. Infect Drug Resist.

[6] Kim S., Lee K.H., Lee J., Lee S.K., Chun Y., Lee J.H., Yoo H.Y. (2023). Efficient recovery strategy of luteolin from agricultural waste peanut shells and activity evaluation of its functional biomolecules. Int. J. Mol. Sci..

[7] Gao A.X., Xia T.C.X., Peng Z.T., Wu Q.Y., Zhu Y., Dong T.T.X., Tsim K.W.K. (2023). The ethanolic extract of peanut shell attenuates the depressive-like behaviors of mice through modulation of inflammation and gut microbiota. Food Res. Int..

[8] Wang T.T., Wang S.K., Huang G.L., Sun G.J. (2012). Luteolin induced-growth inhibition and apoptosis of human esophageal squamous carcinoma cell line Eca109 Cells in vitro. Asian Pac. J. Cancer Prev..

[9] Pu Y.S., Zhang T., Wang J.H., Mao Z.J., Duan B.J., Long Y.B., Xue F., Liu D., Liu S.D., Gao Z.Z. (2018). Luteolin exerts an anticancer effect on gastric cancer cells through multiple signaling pathways and regulating miRNAs. J CANCER.

[10] Sun J.H., Wang Z.D., Chen L., Sun G.J. (2021). Hypolipidemic effects and preliminary mechanism of chrysanthemum flavonoids, its main components luteolin and luteoloside in hyperlipidemia rats. Antioxidants-Basel.

[11] Abidin L., Miljeeb M., Mir S.R., Khan S.A., Ahmad A. (2014). Comparative assessment of extraction methods and quantitative estimation of luteolin in the leaves of Vitex negundo Linn. by HPLC. Asian Pac. J. Trop. Med..

[12] Manzoor M.F., Ahmad N., Ahmed Z., Siddique R., Zeng X.A., Rahaman A., Aadil R.M., Wahab A. (2019). Novel extraction techniques and pharmaceutical activities of luteolin and its derivatives. J. Food Biochem..

[13] Masala V., Jokic S., Aladic K., Molnar M., Casula M., Tuberoso C.I.G. (2024). Chemical profiling and evaluation of antioxidant activity of artichoke (cynara cardunculus var. scolymus) leaf by-products' extracts obtained with green extraction techniques. Molecules.

[14] Fu Y.J., Liu W., Zu Y.G., Tong M.H., Li S.M., Yan M.M., Efferth T., Luo H. (2008). Enzyme assisted extraction of luteolin and apigenin from pigeonpea [Cajanus cajan (L.) Millsp.] leaves. Food Chem..

[15] Zhao H.Y., Wang J.P., Han Y.T., Wang X., Ab Z.S. (2024). Optimization of process conditions for ionic liquid-based ultrasound-enzyme-assisted extraction of resveratrol from Polygonum Cuspidatum. Ultrason. Sonochem..

[16] Karabegovic I.T., Stojicevic S.S., Velickovic D.T., Nikolic N.C., Lazic M.L. (2018). Direct ultrasound-assisted extraction and characterization of phenolic compounds from fresh houseleek (Sempervivum marmoreum L.) leaves. HEMIND.

[17] Dinodia M. (2022). Ionic liquids: environment-friendly greener solvents for organic synthesis. Curr. Org. Synth..

[18] Yang L., Wang H., Zu Y.G., Zhao C.J., Zhang L., Chen X.Q., Zhang Z.H. (2011). Ultrasound-assisted extraction of the three terpenoid indole alkaloids vindoline, catharanthine and vinblastine from Catharanthus roseus using ionic liquid aqueous solutions. Chem. Eng. J..

[19] Wang J.L., Feng J., Xu L., Ma J.P., Li J.L., Ma R., Sun K., Wang Z.B., Zhang H.Q. (2019). Ionic liquid-based salt-induced liquid-liquid extraction of polyphenols and anthraquinones in Polygonum cuspidatum. J. Pharm. Biomed. Anal..

[20] Mohseni M., Mousavi M., Kiani H., Tao Y., Homayoonfal M. (2024). Ionic liquid-based ultrasonic-assisted extraction of L-citrulline from watermelon rind. Waste Biomass Valorization.

[21] Wang J.P., Zhao H.Y., Xue X.X., Han Y.T., Wang X., Sheng Z.L. (2024). Application of ionic liquid ultrasound-assisted extraction (IL-UAE) of lycopene from guava (Psidium guajava L.) by response surface methodology and artificial neural network-genetic algorithm. Ultrason. Sonochem..

[22] Macías-Sánchez M.D., Mantell C., Rodríguez M., de la Ossa E.M., Lubián L.M., Montero O. (2009). Comparison of supercritical fluid and ultrasound-assisted extraction of carotenoids and chlorophyll a from Dunaliella salina. Talanta.

[23] Lajoie L., Fabiano-Tixier A.S., Chemat F. (2022). Water as green solvent: methods of solubilisation and extraction of natural products-past, present and future solutions. Pharmaceuticals-Base.

[24] Giacometti J., Zauhar G., Zuvic M. (2018). Optimization of ultrasonic-assisted extraction of major phenolic compounds from olive leaves (Olea europaea L.) using response surface methodology. Foods.

[25] Cheng J., Li Q.S. (2008). Reliability analysis of structures using artificial neural network based genetic algorithms. Comput. Methods Appl. Mech. Eng..

[26] Wang Y.G., Wang C.L., Xue H.Y., Jin Y.M., Yang M.J., Leng F.F. (2023). Comparative analysis of three kinds of extraction kinetic models of crude polysaccharides from Codonopsis pilosula and evaluate the characteristics of crude polysaccharides. Biomass Convers. Biorefin..

[27] Wang L.L., Bai M.G., Qin Y.C., Liu B.T., Wang Y.B., Zhou Y.F. (2018). Application of ionic liquid-based ultrasonic-assisted extraction of flavonoids from bamboo leaves. Molecules.

[28] Sui X.Y., Liu T.T., Liu J.C., Zhang J., Zhang H.L., Wang H.Y., Yang Y. (2020). Ultrasonic-enhanced surface-active ionic liquid-based extraction and defoaming for the extraction of psoralen and isopsoralen from Psoralea corylifolia seeds. Ultrason. Sonochem..

[29] M.Y. Hou, W.Z. Hu, A.S. Wang, Z.L. Xiu, Y.S. Shi, K.X. Hao, X.S. Sun, D. Cao, R.S. Lu, J. Sun, Ultrasound-assisted extraction of total flavonoids from pteris cretica L.: Process optimization, HPLC analysis, and evaluation of antioxidant activity, Antioxidants-BASEL, 8 (2019) 425.http://dx.doi.org/10.3390/antiox8100425. 10.3390/antiox8100425 PMC6826651 31554157

[30] Zhou Y., Wu D.T., Cai P.F., Cheng G.F., Huang C.B., Pan Y.J. (2015). Special effect of ionic liquids on the extraction of flavonoid glycosides from chrysanthemum morifolium ramat by microwave assistance. Molecules.

[31] Xu J.L., Wang W.C., Liang H., Zhang Q., Li Q.Y. (2015). Optimization of ionic liquid based ultrasonic assisted extraction of antioxidant compounds from Curcuma Longa L. using response surface methodology. Ind. Crops Prod..

[32] Tang M.Y., Cao J.X., Wu Z.X., Ding Y., Ye Q., Li J.L. (2024). Separation of ginsenosides from Panax notoginseng intensified with ionic liquids. Ind. Crops Prod..

